# Supplementary material for: Measuring electric-acoustic heterodyning in piezoelectric materials
Source: Commun Phys. 2025 Dec 21;9(1):26. doi: 10.1038/s42005-025-02457-8 (PMC12823382; doi:10.1038/s42005-025-02457-8)
Supplement: Supplementary file 2 — Supplemental Material PDF [file 42005_2025_2457_MOESM2_ESM.pdf]

Measuring Electric-Acoustic Heterodyning in Piezoelectric Materials

Tomasz Karpisz[1,2], Robert L. Lirette[1,2], Aaron M. Hagerstrom[1], Nathan D. Orloff[1], Angela C. Stelson[1]

Many electrically active devices rely on nonlinear signal mixing (heterodyning) between two electrical signals. Heterodyning between electric and acoustic signals can allow for active control of typically passive components such as transmission lines, acoustic resonators, and electrical resonators built from piezoelectric materials. However, there are few techniques to characterize the nonlinear properties of materials that lead to heterodyning between electric and acoustic signals. Here we demonstrate a proof-of-concept microwave interferometer measurement setup that uses electromagnetic and acoustic waves to measure nonlinear second-order mixing from electrical and acoustic signals in a piezoelectric material. The sum and difference frequencies of signal mixing can be detected in the electromagnetic spectrum in our measurement. We show the effect of frequency and power of the fundamental signals on the mixing products. We additionally characterize the heterodyne signal to show that it is electric-acoustic in nature, versus purely electric. Characterizing nonlinear electric-acoustic properties is important to the development of next generation piezoelectric materials models and devices.

[1] National Institute of Standards and Technology, 325 Broadway Boulder, Colorado 80305, USA. [2]University of Colorado Boulder, Libby Dr, Boulder, Colorado 80302, USA Email: tomasz.karpisz@nist.gov, Phone: (303) 497-6864

Supplementary Methods

Certain commercial equipment, instruments, or materials are identified in this paper in order to specify the experimental procedure adequately. Such identification is not intended to imply recommendation or endorsement by the National Institute of Standards and Technology, nor is it intended to imply that the materials or equipment identified are necessarily the best available for the purpose.

Supplementary Note 1: Finite element simulations of coplanar waveguide

In paper we present theoretical loss (α) and phase constant (β) of electromagnetic wave propagating in CPW with sample on top at Fig. 1 (a). We use those values to predict interferometer transmission. Values of α and β are simulated with a use of finite element simulation software. We assume a quasi-TEM wave propagating in coplanar waveguide (CPW) with a PZT-4 sample and acoustic transducer on top (similar to Fig. 1(b) of the manuscript). The simulation model reflects dimensions and materials used in experimental part of that paper. The substrate material dielectric constant it 3.8 and loss tangent is 0.0027 measured at 10 GHz, as provided by manufacturer. The measured thickness of the substrate is 1.250 mm, the CPW central conductor width is 400 μm, the ground plane widths are 125 μm and the gap between the center conductor and ground planes is 300 μm. The dielectric properties of the PZT-4 block depends on the processing of the PZT material and varies with temperature and frequency but considering refs [1–4] we used values of relative permittivity ($\varepsilon_r = 1400$) and loss tangent ($\tan(\delta) = 0.01$) for the simulation.

Supplementary Note 2: Electrostriction in materials

To illustrate the materials physics that generates electric-acoustic and second order nonlinear mixing in ferroelectric materials, we can formulate the Gibbs free energy Supplementary Equation (1) using Devonshire theory[5,6]. In Einstein summation notation, the Gibbs free energy is:

$$\mathbf{G} = G_0 - \frac{1}{2}\varepsilon_{ij}\mathbf{E_i}\mathbf{E_j} + \frac{1}{2}c_{ijkl}\mathbf{x_{ij}}\mathbf{x_{kl}} - e_{ijk}\mathbf{E_i}\mathbf{x_{jk}} + \frac{1}{2}m_{ijkl}\mathbf{E_i}\mathbf{E_j}\mathbf{x_{kl}} \quad \text{(Se1)}$$

where ε_{ij} is the permittivity, c_{ij} is the elasticity, e_{ij} is the piezoelectric coefficient and m_{ijk} is the electrostriction coefficient. E_i are applied electric field in directions i, j, k, and x_i is induced strain from the acoustic signal in the material in directions i, j, k in Cartesian coordinates. In equilibrium, the displacement field is

$$\mathbf{D_i} = -(\partial\mathbf{G}/\partial\mathbf{E_i})_X \quad \text{(Se2)}$$

Under the assumption that $\varepsilon_{ij} = \varepsilon_{ji}$, $m_{ijk} = m_{jik}$, we arrive at the following description for the displacement field:

$$\mathbf{D}_i = (\varepsilon_{ij} - m_{ijkl}\mathbf{x_{kl}})\mathbf{E_j} + e_{ijk}\mathbf{x_{jk}} \quad \text{(Se3)}$$

In Supplementary Equation (3), the displacement field generated by electrostriction m_{ijk} is proportional to both the electric field and the strain. We interpret this nonlinear contribution to the displacement field as the dominant source of the mixing product signal at frequencies $f_1 \pm f_a$[7].

Supplementary Note 3: Electromagnetic coupling experiment

As explained in the main manuscript, a piezoelectric sample has electrical, acoustic and electric-acoustic nonlinear properties. In this experimental configuration, where electrical signals are transduced into the acoustic

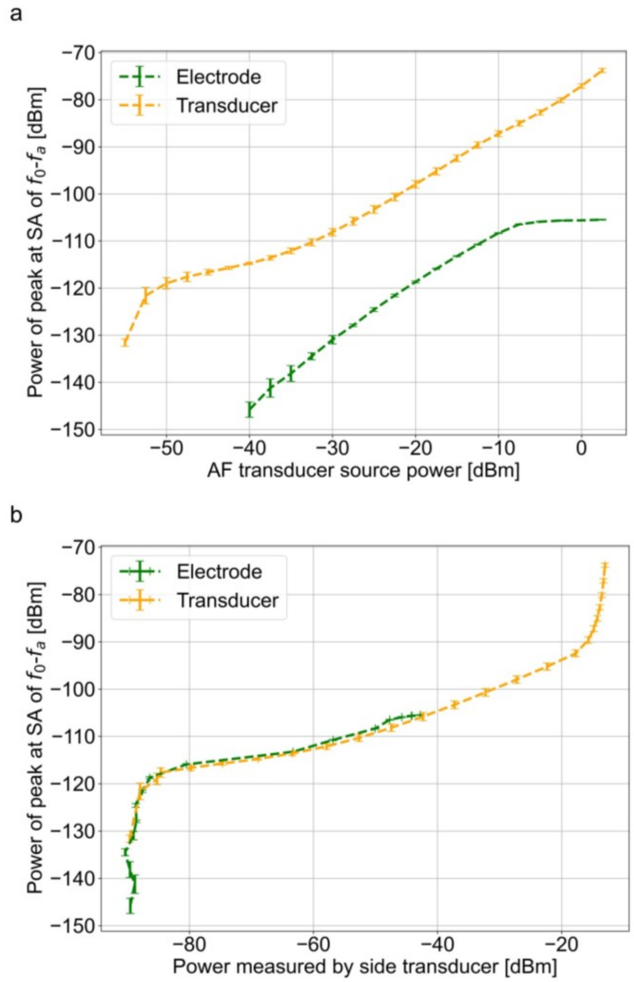

Fig. S1 (a) **Power level of nonlinear mixing product measured by spectrum analyzer (SA) as a function of acoustic frequency (AF)** source power for acoustic transducer (green line) and electrode (orange line). (b) Power level of nonlinear mixing product measured by SA as a function of acoustic signal power in piezoelectric lead zirconium titanate (PZT) bulk measured by side transducer (orange line) and electrode (green line).

domain near the sample material, it is important to consider the possibility of electrical heterodyning as a contribution to the nonlinear mixing product $A(f_1 \pm fa)$. These contributions to the signal would result from the electrical signal from AF source coupling into the PZT and giving rise to mixing product signals via the second order electrical nonlinear coefficient. To test this hypothesis, we performed a series of experiments designed to isolate the effects of the purely electromagnetic contributions.

First, we fabricated a dummy electrode with similar dimensions to our acoustic transducer. Our transducer was made of 1 x 15 x 20 mm PZT material, coated with silver on the top and bottom to form a parallel plate capacitor. For our dummy electrode, we used an acrylic block of similar dimensions (1.2 x 16 x 22 mm) coated with silver in a similar configuration. By using a non-piezoelectric material, we mimicked the electric field radiation pattern of the electrode without transducing a signal into the acoustic domain. Fig. S2 (b) shows the impact of using the electrode in the measurement setup compared to the acoustic transducer. The electrode still produces a signal $A(f_1 \pm fa)$, but the amplitude is reduced by at least 20 dBm. Note that only one nonlinear mixing product $A(f_1 - fa)$ is shown since the difference between both of the two peaks power have been shown on Fig. 4, and the goal of this figure is to demonstrate the nature of nonlinear effects. This result shows that the electric field generated by the electrode configuration is coupling into the sample and the experimental setup, but at a much lower level than the acoustically transduced signal.

Supplementary Note 4: Pre-amplifier measurements
To understand why the power measured by the spectrum analyzer (SA) with the preamplifier on and off does not exactly match in Fig. 3 (b) it is crucial to perform one additional experiment. In this experiment, the microwave source (MS) was directly connected to the spectrum analyzer and a set of measurements were done. Power on the spectrum analyzer was measured as a function of different frequencies and MS power levels for the preamplifier turned on and off. The difference between preamplifier setting can be seen in Fig. 5. That difference can reach 5 dB for power as low as -41 dBW. At the frequency the measurements were done, there was a difference of 0.25 dB even for the lowest tested power levels. This explains the difference of power levels at Fig. 3 (b).

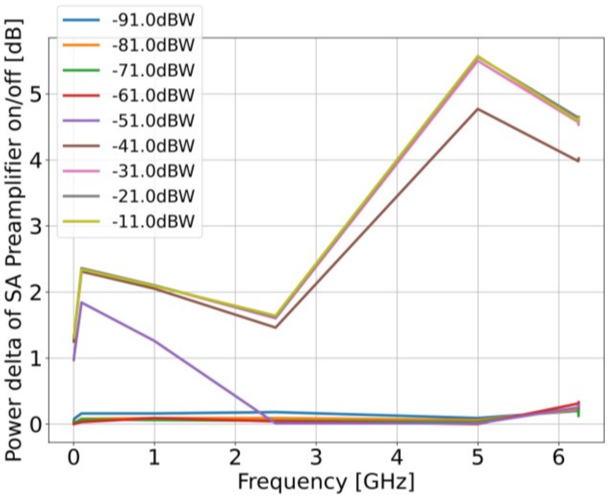

Fig. S2 **Preamplifier Influence.** Difference between power measured at spectrum analyzer (SA) for preamplifier turned on and off for a different frequency and power level of microwave source.

Supplementary Note 5: Interferometer Noise Floor
The main purpose of Figure 2. c) of the main manuscript is to show HF and nonlinear mixing signal peaks at $A(f_1)$ and $A(f_1 \pm f_a)$ on the interferometer background characteristics. The noise floor level is combination of number of factors. First is the noise floor level of HF Source which for power used for experiments is around -97 dBm at and $A(f_1 \pm f_a)$, and gets higher closer to $A(f_1)$. That noise floor level can be lowered using interferometer. In theory at the frequency f_{null} where interferometer has its null the cancelation is full and the transmission level is $-\infty$ dBm (see Fig 1. c) yellow line). In practice the measurement setup noise floor is limited by used detector and its settings. To achieve minimal noise floor for used spectrum analyzer it is necessary to use narrow bandpass IF filter setting. Measurements where the data has been collected for Fig 3 and 4 has been made for 2 kHz span, 10 Hz IF filter setting and averaging of 16, that resulted in 3.3 second sweep time with spectrum analyzer minimal possible noise floor level of around -140 dBm while preamplifier was turned on. That value can be seen as a limit on Fig 4. a) where the preamplifier was used at lowest HF source power. Purpose of Fig 2 c) was to show all three peaks $A(f_1)$ and $A(f_1 \pm f_a)$ therefore we choose span of 5 MHz chosen. If the same spectrum analyzer settings were used, we would need 1 hour and 23.5 second for that measurement. Since Fig 2 c) goal was to show all measured peaks, we chose IF filter bandwidth of 1 kHz which resulted in 23.1 second sweep time and minimal cancelation artificially increased to -110 dBm (see Fig 2. c)). Finally, preamplifier could not be turned off for that figure in a presence of high power $A(f_1)$ signal that could damage spectrum analyzer receiver, which limited noise floor level to -110 dBm.

Below we present a table with noise floor values for two cases. 1) f_1 set as the fundamental frequency provided by the HF source frequency) to be f_{null} (interferometer null), and 2) f_{null} set as the mixing product frequency $f_1 \pm f_a$. In First column we put noise floor level at $A(f_1 \pm f_a)$ for setup without interferometer where the noise floor is set by HF source, second column shows noise floor levels at $A(f_1 \pm f_a)$ with a use of interferometer, and third column shows noise floor at $A(f_1)$ with interferometer. Note that for the 1) case values in columns 2 and 3 are the same while for case 2) values in columns 3 and 4 are the same. It is due to frequency of acoustic transducer being equal to $f_a = 2.2$ MHz. If the f_a frequency would be lower, the nonlinear mixing peaks

$A(f_1 \pm f_a)$ would be closer in frequency to the $A(f_1)$ peak, and for the case 1), value in column 2 would be higher as the noise floor level gets higher the closer in frequency it is to $A(f_1)$ while value at the column 3 would be lower as the cancelation of the interferometer gets bigger closer to the f_{null} frequency. As a result, use of interferometer in that experiment allowed us to lower noise floor level caused by nonlinearities of HF source by 43 dB to the level of -140 dBm.

Table S1. Noise floor for different experimental configurations with and without cancellation from the interferometer.

Interferometer null frequency f_{null}	Noise floor at $A(f_1 \pm f_a)$ without cancellation	Noise floor at $A(f_1 \pm f_a)$ with cancellation	Noise floor at $A(f_1)$ with cancellation
$A(f_1)$	~97 dBm	~97 dBm	-140 dBm
$A(f_1 \pm f_a)$	~97 dBm	-140 dBm	~97 dBm

Supplementary References

1. Hooker, M. W. *Properties of PZT-Based Piezoelectric Ceramics Between -150 and 250 C*. (1998).
2. Wang, W., Liang, R., Zhou, Z., Zhang, Y. & Dong, X. Defect engineering for reduced large AC signal dielectric loss of PZT-based hard piezoelectric ceramics. *Journal of the American Ceramic Society* 105, 279–291 (2022).
3. Li, G. *et al.* Investigation of High-Power Properties of PIN-PMN-PT Relaxor-Based Ferroelectric Single Crystals and PZT-4 Piezoelectric Ceramics. *IEEE Trans Ultrason Ferroelectr Freq Control* 67, 1641–1646 (2020).
4. Rossi, J. O., Neto, L. P. S. & Yamasaki, F. S. Operation of dielectric nonlinear transmission lines based on ceramic PZT slabs. in *2014 IEEE International Power Modulator and High Voltage Conference (IPMHVC)* 217–220 (2014). doi:10.1109/IPMHVC.2014.7287247.
5. Uchino, K. ELECTROSTRICTIVE MATERIALS. in *Encyclopedia of Vibration* (ed. Braun, S.) 475–490 (Elsevier, Oxford, 2001). doi:https://doi.org/10.1006/rwvb.2001.0078.
6. Mangeri, John, 'Computational Design of Multifunctional Nanodielectrics' (2017). Doctoral Dissertations. 1540. https://opencommons.uconn.edu/dissertations/1540.
7. Hagerstrom, A. M. *et al.* Measurements of Nonlinear Polarization Dynamics in the Tens of Gigahertz. *Phys Rev Appl* 13, 44026 (2020).
